# Allocating Antiretrovirals in South Africa: Using Modeling to Determine Treatment Equity

**DOI:** 10.1371/journal.pmed.0020155

**Published:** 2005-06-28

**Authors:** David P Wilson, Sally M Blower

**Affiliations:** University of CaliforniaLos Angeles, CaliforniaUnited States of America

Recently *PLoS Medicine* published our paper entitled “Designing Equitable Antiretroviral Allocation Strategies in Resource-Constrained Countries” [1]. We were disappointed to find that the editorial perspective written by the World Health Organization (WHO) ethicists regarding our paper [2] was based upon a substantial misunderstanding of our novel quantitative analyses and our important results. Hence, they misunderstood the significance of the health-policy implications of our results. Thus, we wish to correct the record.

Firstly, Capron and Reis [2] misunderstood our quantitative analyses. They stated that “Wilson and Blower developed a mathematical model that could inform policy-makers' decisions regarding the optimal distribution of treatment sites to ensure equal access by all individuals infected with HIV.” However, our model does not determine the optimal distribution of treatment sites. As we clearly state in our paper [1] (and is also stated in the synopsis [3]), we developed a model that policy makers can use to make decisions regarding how to achieve the optimal allocation of scarce antiretrovirals among the available health-care facilities (HCFs) if the objective is to ensure treatment equity. We also calculated how the optimal allocation of antiretrovirals would vary if the number of HCFs utilized increased and/or the size of the catchment area that each HCF services increased [1]. Thus, we took the treatment sites (i.e., HCFs) as given, and we used their specific spatial location in South Africa as inputs to our model in order to determine optimal antiretroviral allocation strategies under a variety of conditions.

Secondly, Capron and Reis [2] misunderstood our important results. They stated that “applying this tool to the South-African province of KwaZulu–Natal, Wilson and Blower were able to confirm mathematically the intuitive assumption that using a maximum number of centers, at the least possible distance from most affected populations, would lead to the greatest fairness in the geographical distribution of ART [antiretroviral therapy].” We agree that if these had been our results, they would have been trivial and obvious. However, Capron and Reis [2] did not discuss our actual results: we determined how to decide how many drugs to allocate to each of the available HCFs in order to achieve an optimal allocation if the objective is to ensure treatment equity. This is a very complex problem and the antiretroviral allocation strategies that we calculated (by using our model) to be optimal are very complex (see Figure 3 in our paper, which graphically shows the proportion of drugs that should be allocated to each of the available HCFs). Furthermore, we also determined what catchment area each HCF should service; specifically, we calculated that each HCF should serve (if the objective is to achieve treatment equity) a catchment area of 40–60 km. Thus, our results demonstrate (to our knowledge for the first time) that patients infected with HIV will have to travel extremely large distances (i.e., 40-–60 km) in order to receive antiretrovirals, if the objective is to achieve treatment equity in South Africa. We stress that currently it is unknown what the actual size of the catchment area is around HCFs in South Africa. Catchment areas may in fact be very small. Thus, we suggested [1] that a primary goal should be to obtain empirical data of the distances that patients in South Africa are willing (or able) to travel in order to receive antiretrovirals. We have been the first to provide a quantitative assessment of the necessary size of the catchment area, and our results have identified that there is an urgent need to collect these critical data for quantifying the size of the catchment areas around HCFs. We have determined that the size of the catchment area will be a critical component in the ability to achieve treatment equity in South Africa. We also compared the optimal antiretroviral allocation strategies that we calculated with the current plan of the South African government for allocating antiretrovirals [4], and we determined that the current antiretroviral allocation strategies in South Africa will not achieve treatment equity. Taken together, our quantitative results are novel and controversial, providing important quantitative insights into a complex public-health problem.

We applaud the ambitious “3 by 5” WHO target for the antiretroviral rollout. However, the WHO has not yet devised a quantitative policy for determining how to allocate antiretrovirals in situations where the demand for drugs greatly exceeds the supply [5]. Health-policy officials in each country will have to make these important and difficult decisions, and they will all make different decisions based upon what objectives they wish to optimize and prioritize. There are a multitude of factors to consider (these factors are well described in the recent *Institute of Medicine* report [6]). We stress that the alternative to a quantitative rational approach for allocating scarce resources is an ad hoc approach, which is how the scarce supply of antiretrovirals is currently being distributed in many resource-constrained countries. Our operations research modeling approach is based upon spatial heterogeneity in the distribution of HCFs in South Africa and the spatial heterogeneity of the HIV-infected population. The most important “real world” result is that we show that what the South African government is currently doing is inequitable. We show them how to achieve equity, if they wish to do so. We hope that our novel approach for deciding how to allocate antiretrovirals will be of use to the WHO and also to the relevant authorities in the many resource-constrained countries who will soon have to make very difficult decisions as to who lives and who dies. Our analysis is to our knowledge the first analysis to show how a rational and scientific solution can be reached for deciding how to allocate a limited amount of antiretrovirals, if the goal is to achieve treatment equity. Clearly, other goals must be taken into consideration (and our model can be modified to include these other goals); however, we hope that treatment equity will be a very high priority during the antiretroviral rollout that is just beginning.
